# Effects of calorie restriction on the lifespan and healthspan of POLG mitochondrial mutator mice

**DOI:** 10.1371/journal.pone.0171159

**Published:** 2017-02-03

**Authors:** Shinichi Someya, Gregory C. Kujoth, Mi-Jung Kim, Timothy A. Hacker, Marc Vermulst, Richard Weindruch, Tomas A. Prolla

**Affiliations:** 1 Department of Aging and Geriatric Research, University of Florida, Gainesville, Florida, United States of America; 2 Department of Neurological Surgery, University of Wisconsin, Madison, Wisconsin, United States of America; 3 Department of Medicine, University of Wisconsin, Madison, Wisconsin, United States of America; 4 Center for Mitochondrial and Epigenomic Medicine, University of Pennsylvania, Philadelphia, Pennsylvania, United States of America; 5 Veterans Administration Hospital, Geriatric Research, Education and Clinical Center, University of Wisconsin, Madison, Wisconsin, United States of America; 6 Departments of Genetics & Medical Genetics, University of Wisconsin, Madison, Wisconsin, United States of America; RIKEN Brain Science Institution, JAPAN

## Abstract

Mitochondrial DNA (mtDNA) mutations are thought to have a causative role in age-related pathologies. We have shown previously that mitochondrial mutator mice (*Polg*^D257A/D257A^), harboring a proofreading-deficient version of the mtDNA polymerase gamma (POLG), accumulate mtDNA mutations in multiple tissues and display several features of accelerated aging. Calorie restriction (CR) is known to delay the onset of age-related diseases and to extend the lifespan of a variety of species, including rodents. In the current study we investigated the effects of CR on the lifespan and healthspan of mitochondrial mutator mice. Long-term CR did not increase the median or maximum lifespan of *Polg*^D257A/D257A^ mice. Furthermore, CR did not reduce mtDNA deletions in the heart and muscle, accelerated sarcopenia, testicular atrophy, nor improve the alterations in cardiac parameters that are present in aged mitochondrial mutator mice. Therefore, our findings suggest that accumulation of mtDNA mutations may interfere with the beneficial action of CR in aging retardation.

## Introduction

Calorie restriction (CR) is the most robust non-genetic intervention to consistently extend lifespan in a variety of species [1]. CR also reduces risks for a variety of age-associated diseases, including diabetes, sarcopenia, cancer, cardiovascular diseases, and hearing loss in rodents and humans [1–3]. Furthermore, CR reduces the levels of reactive oxygen species (ROS) and associated oxidative damage, and mtDNA deletions in multiple tissues [1–2, 4–5]. Consistent with these reports, long-lived GH (growth hormone)-deficient mice display CR-like anti-aging effects, including increased expression of antioxidant enzymes and stress response genes in muscle cells and fibroblasts [2], reduced body size and extended median lifespan [6]. The mitochondrial theory of aging postulates that ROS generated inside the mitochondria damage key mitochondrial components such as mitochondrial DNA (mtDNA), resulting in mitochondrial dysfunction [7]. This in turn leads to energy depletion in multiple tissues and eventually to aging symptoms. Accordingly, CR is thought to slow the rate of aging or to reduce risks for many age-associated diseases through the protection of mitochondrial macromolecules, including mtDNA [1, 5, 8–10]. In the current study, we investigated the effects of CR on the healthspan and lifespan of mice that express a proofreading-deficient version of mtDNA polymerase gamma (*Polg*^D257A/D257A^) and display elevated mtDNA mutation frequencies throughout their tissues and accelerated aging phenotypes [11–14].

## Materials and methods

### Animal husbandry & diets

Generation and characterization of *Polg*^D257A/D257A^ mice have been previously described [11]. Male *Polg*^*+/D257A*^ mice were backcrossed to C57Bl/6J female mice for four generations and N4 heterozygous mice were subsequently intercrossed to generate the wild type, heterozygous, and homozygous littermates used in this study. Mice were housed individually (12 hour light/dark cycle), placed on control (84 kcal/week; Teklad #91349) or calorie-restricted (63 kcal/week; Teklad #91351) semi-purified diets at 2–3 months of age and maintained on these agar gelatin diets for the duration of the study; there were no statistically significant differences between mean diet starting ages among genotype-diet groups. These dietary formulations were isocaloric (4.1 kcal/g) and had similar fat proportions (30–31%); the CR diet had an enriched mineral (AIN-76) formulation however, such that vitamin and mineral consumption was similar between the two diets at their final feeding amounts [15]. Feeding followed a Monday (2/7 weekly allowance), Wednesday (2/7), Friday (3/7) schedule.

All mice used for the survival studies were allowed to live out their life (natural death). However, any mice that appeared ill or chronically distressed were euthanized before natural death by CO_2_ inhalation. All mice were examined daily for signs of ill health. Specific criteria were: loss of mobility, paralysis, lethargy or unresponsiveness, palpable tumor masses not to exceed 1.5 cm in diameter, body weight loss of 15% after achieving a steady weight on study, ulcerative dermatitis, and refusing food or water for more than 2 days. The lifespan, cardiac function and sarcopenia studies were performed under protocols approved by Animal Care and Use Committees of the University of Wisconsin-Madison and the William S. Middleton Memorial Veterans Administration Hospital, while the dietary restriction study for the mtDNA mutation frequency measurements was performed in accordance with protocols approved by the University of Florida Institutional Animal Care and Use Committee.

### Body weight and tissue weight

The body weight of the mice was measured every month from 2 months of age until spontaneous death. The weight of tissues (muscle, testes, and heart) of the mice was measured at 13–15 months of age.

### Randam capture mutation assay

Mitochondria were resuspended in lysis buffer containing 10 mM Tris—HCl, 150 mM NaCl, 5 mM EDTA, 0.2 mg/ml proteinase K and 0.5% SDS and lysed at 55°C for 30 min. DNA was then isolated by 2 phenol—chloroform-isoamyl alcohol extractions, followed by a single chloroform extraction and ethanol precipitation. The isolated mtDNA was digested in a unique TaqI buffer from Thermo Scientific (ER0671) by adding an additional 100 U of TaqI enzyme (NEB, R0149M) every hour over a 5-hour time span. The mtDNA was then cleaned up with an oligo-concentrator kit from Zymogen and re-digested for another 5 hours under the same conditions. Finally, mutations were detected inside a TaqI restriction site located at bp 634 of the mitochondrial genome, and two sites that are prone to deletions (site 1 and site 3), using SYBR green based qPCR and previously published protocols [13–14]. The following primers were used for mutation detection: Control primer forward: TCGGCGTAAAACGTGTCAAC;

Control primer reverse: CCGCCAAGTCCTTTGAGTTT;

Taq634 primer forward: ACTCAAAGGACTTGGCGGTA;

Taq634 primer reverse: AGCCCATTTCTTCCCATTTC;

Deletion primer site 1 forward: AGGCCACCACACTCCTATTG;

Deletion primer site 1 reverse: AATGCTAGGCGTTTGATTGG;

Deletion primer site 3 forward: ACGAAATCAACAACCCCGTA;

Deletion primer site 3 reverse: AATGCTAGGCGTTTGATTGG.

### Cardiac function

Echocardiography was performed using an Acuson Sequoia (Siemens) ultrasonograph with a 15-MHz transducer. For acquisition of two dimensional guided M-mode images at the tips of papillary muscles and Doppler studies, mice were sedated by IP administration of 100 mg/kg ketamine and maintained on a heated platform in a left lateral decubitus position. All images were saved to an on-board optical disk as previously described [16].

End diastolic and systolic left ventricular (LV) diameter as well as anterior and posterior wall (AW and PW respectively) thicknesses were measured on line from M-mode images using the leading edge-to-leading edge convention. All parameters were measured over at least three consecutive cardiac cycles and averaged. Left ventricular fractional shortening was calculated as [(LV diameter diastole—LV diameter systole)/LV diameter diastole] x 100 and LV mass was calculated by using the formula [1.05 x ((Posterior Wall diastole + Anterior Wall diastole + LV diameter diastole)^3^ –(LV diameter diastole)^3^)]. Relative wall thickness was calculated as 2 x Posterior wall diastole/LV diameter diastole. Heart rate was determined from at least three consecutive intervals from the pulse wave Doppler tracings of the LV outflow tract. Isovolumic relaxation time was measured as the time from the closing of the aortic value to the opening of the mitral value from pulse wave Doppler tracings of the LV outflow tract and mitral inflow region. The same person obtained all images and measures.

### Survival and statistical analysis

Kaplan-Meier survival analysis was performed with GraphPad Prism 6 (La Jolla, CA) and survival curves were compared using the log rank (Mantel-Cox) test. Median and 90^th^ percentile values were calculated from the earliest time points at which the survival proportions were less than or equal to 0.5 or 0.1, respectively. Mean survival times are calculated as the area under the survival curves.

Age and weight data were compared by one-way ANOVA, followed by Šídák’s test for selected multiple comparisons to generate multiplicity-adjusted P values with family-wide alpha = 0.5. Echocardiography data were compared by t-tests and were reported without adjusting for multiple tests.

## Results

**Fig 1:**
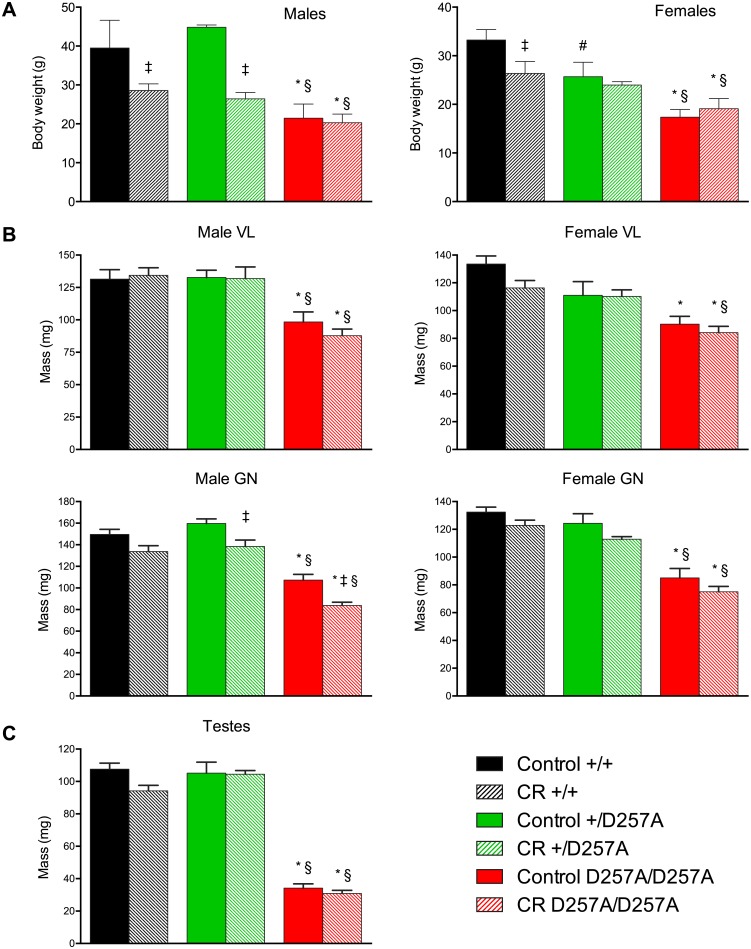
Body weight, skeletal muscle mass, and testis weight of mitochondrial mutator mice. Body weights (A), vastus lateralis (VL) and gastrocnemius (GN) muscle mass (B) of 13–16 months old +/+, +/D257A, and D257A/D257A males (left) and females (right) under control diet or calorie restricted conditions. (C) Testes weights of 10–18 months old +/+, +/D257A, and D257A/D257A males under control diet or calorie restricted conditions. ‡P < 0.05 control diet vs CR diet within genotype. n = 8–16. *P < 0.05 +/+ vs D257A/D257A within diet. #P < 0.05 +/+ vs +/D257A within diet. §P < 0.05 +/D257A vs D257A/D257A within diet. +/+ = *wild-type*, +/D257A = *Polg*^+/D257A^, and D257A/D257A = *Polg*^D257A/D257A^.

We and others have previously shown that D257A/D257A mice display a range of premature aging phenotypes beginning at ~9 months of age, including body weight loss, loss of bone mass, and reduced lifespan [11–12]. To investigate the effects of CR on the healthspan and/or lifespan of mitochondrial mutator mice, we reduced the calorie intake of *wild-type* (+/+), *Polg*^+/D257A^ (+/D257A), and *Polg*^D257A/D257A^ (D257A/D257A) mice in the C57BL/6 background to 75% (a 25% CR) of that fed to control diet (CD) mice starting in early adulthood (2 months of age), and this diet regimen was maintained until the animals reached middle-age (13–16 months of age) or spontaneous death of the animals occurred. CD mice were fed a control diet (Teklad, Madison, WI) and received 84 kcal/week of the diet, while CR mice were fed a restricted diet (Teklad, Madison, WI) of 63 kcal/week. The restricted diet was enriched in protein, vitamins, and minerals to avoid malnutrition. This is a well-established CR protocol that extends lifespan in inbred mouse strains, including the C57BL/6 strain [15]. We first confirmed that this CR diet regimen resulted in a significant reduction of body weight in middle-aged +/+ males and females (Fig 1). CR also resulted in a significant reduction of body weight in middle-aged +/D257A males, but not in middle-aged +/D257A females. Although middle-aged D257A/D257A mice weighed less than their +/+ or +/D257A counterparts, CR did not further reduce the body weight of D257A/D257A males or females. We note that the specific cause of reduced weight in D257A/D257A mice is unknown, although it is likely to be a combination of reduced adipose tissue and muscle mass in these animals, as well as degeneration of other organs. These phenotypes become progressively worse with age [11]. Thus, if CR reduced accelerated aging in D257A/D257A mice, we speculate that the weight of POLG mice should be more similar to that of +/+ animals at middle age.

**Fig 2:**
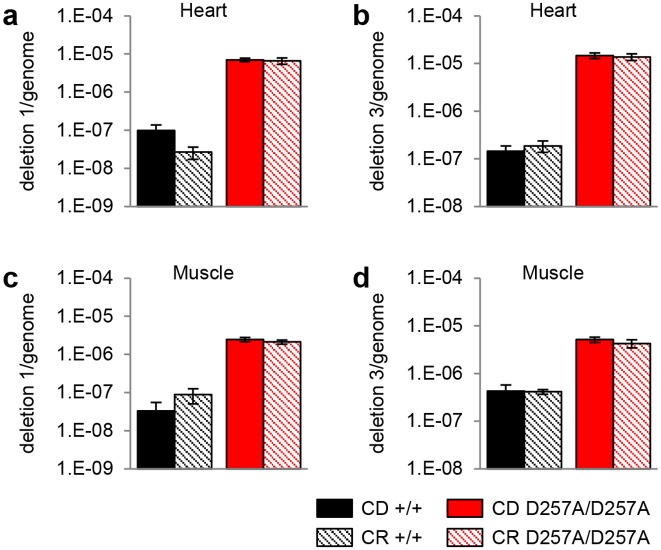
Mitochondrial deletion load in WT and D257A/D257A mice. mtDNA deletions 1 and 3 in the heart (A) and muscle (B) of 13–16 months old +/+ and D257A/D257A mice under control diet or calorie restricted conditions. n = 4. +/+ = *wild-type*, D257A/D257A = *Polg*^D257A/D257A^.

We and others have shown previously that mtDNA deletions accumulates at an accelerated rate in the heart, brain, liver, and intestine of middle-aged mitochondrial mutator mice, while mitochondrial DNA deletions accumulate significantly in the brain and heart of old C57BL/6 mice [11–14]. In contrast, CR is known to reduce mtDNA deletions in the brain and liver of aged rats [8–10]. Next, we investigated the effects of CR on mtDNA deletions in the heart and muscle tissues of 13–16 months old +/+ D257A/D257A mice under control diet or calorie restricted diet. We first confirmed that middle-aged D257A/D257A mice displayed a 71-fold increase in mtDNA deletions at site 1 and a 102-fold increase in mtDNA deletions at site 3 in the heart when compared to age-matched +/+ mice under control diet conditions (Fig 2). In the muscles, middle-aged D257A/D257A mice also displayed a 74-fold increase in mtDNA deletions at site 1 and a 12-fold increase in mtDNA deletions at site 3 (Fig 2). However, CR did not reduce the mtDNA deletions at site 1 or 3 in the heart or muscle of middle-aged D257A/D257A mice. We note that normally-aged mice typically display increased mtDNA deletions at 24–30 months of age [13, 17].

Aging is characterized by loss of muscle mass or sarcopenia [8]. We have previously reported that D257A/D257A mice display accelerated sarcopenia [11]. Consistent with our previous report, middle-aged D257A/D257A males and females displayed a significant reduction in the weights of both vastus lateralis and gastrocnemius muscles relative to +/+ mice under control diet conditions (Fig 1). CR did not ameliorate this loss of muscle mass. There were no differences in the weights of vastus lateralis muscles between control diet and calorie-restricted D257A/D257A middle aged males and females (Fig 1). There was a small decline in the weights of gastrocnemius muscles between control diet and calorie-restricted D257A/D257A middle aged males, and no difference in females (Fig 1). Furthermore, CR was unable to prevent testicular atrophy in the D257A/D257A mice (Fig 1) that is associated with loss of spermatogonia [11–12]. We note that normally-aged C57BL/6 mice typically display muscle atrophy around 24–30 months of age [18–19].

Age-related decline of heart function is a hallmark of aging across multiple species [15]. We investigated the effects of CR on cardiac function in 13–16 months old +/+ and D257A/D257A mice (combined sexes) under control diet or calorie restricted diet regimens using echocardiography. Compared to +/+ mice, middle-aged D257A/D257A mice under control diet conditions displayed a significant increase in heart rate, left ventricular mass/body weight, stroke volume, and cardiac index as well as a decreased isovolumic relaxation time/heart rate (a measure of diastolic function) (Table 1). In contrast, there were no differences in any cardiac functional parameters between control diet and calorie-restricted D257A/D257A mice, indicating that the CR diet did not slow the development of cardiac phenotypes. We note that normally-aged C57BL/6 mice typically display cardiac muscle atrophy at >24 months of age [20].

**Table 1:** Echocardiography of 13–16 months old wild-type and *Polg*^D257A/D257A^ mice on control and calorie-restricted diets.

		Control diet				CR diet					
		+/+		D257A/D257A		+/+		D257A/D257A			
Measurement	Units	Mean	SD	Mean	SD	Mean	SD	Mean	SD	P^a^	P^b^
Age	mo	14.8	1	13.9	0.8	15	0.9	14.6	0.8	0.167	0.206
Body Weight	g	35.3	4.5	20.9	3.4	27.9	2.5	19.6	1.4	0.0001	0.397
Heart Rate	bpm	501	46	597	59	473	87	543	48	0.01	0.124
LVID;d	mm	3.74	0.26	3.75	0.31	3.62	0.3	4.03	0.31	0.939	0.165
LVPW;d	mm	0.89	0.09	0.94	0.06	0.85	0.11	0.91	0.12	0.291	0.638
LVAW;d	mm	0.89	0.14	0.9	0.09	0.82	0.08	0.92	0.1	0.867	0.755
FS	%	36.3	3.1	39.2	4	37.3	5.7	37.7	7.5	0.186	0.702
LV Mass	mg	98	20.6	102.4	15.3	84.7	13.9	114.4	16.9	0.695	0.253
LV mass/Body weight	mg/g	2.81	0.63	4.95	0.7	3.04	0.46	5.88	0.99	0.0003	0.113
IVRT	ms	16.1	1.8	13.9	1.9	16.9	2	14.3	2.9	0.068	0.817
IVRT/Heart rate	ms/bpm	0.032	0.004	0.023	0.002	0.038	0.012	0.027	0.005	0.001	0.124
Ao VTI	cm	4.18	0.58	3.61	0.66	3.89	0.93	5.03	1.29	0.146	0.055
Stroke Volume	ul	22.9	6.1	31.5	2.7	22.2	4.2	36.5	13.4	0.016	0.439
Cardiac Index	ul/min/g	335	121	918	198	386	130	1006	358	0.0001	0.639

Sample sizes: +/+ Control (N = 7); D257A/D257A Control (N = 5); +/+ CR (N = 7); D257A/D257A CR (N = 6).

^a^control diet +/+ vs control diet D257A/D257A comparison;

^b^control diet D257A/D257A vs calorie-restricted D257A/D257A comparison.

P values are calculated by t-test and are uncorrected for multiple tests. LV, left ventricular; ID, interior diameter; d, diastole; PW, posterior wall; AW, anterior wall; FS, fractional shortening; IVRT, isovolumic relaxation time; Ao, aortic; VTI, velocity time integral; +/+, wild type; CR, caloric restriction.

**Fig 3:**
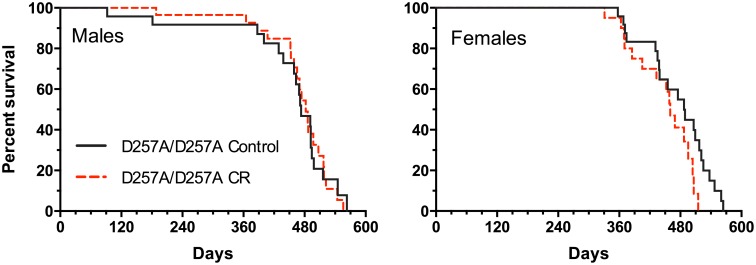
Kaplan-Meier survival curves of D257A/D257A males and females. Survival curves of control diet (solid black line) and calorie restricted (red dashed line) D257A/D257A males (left) and females (right). n = 23–29. D257A/D257A = *Polg*^D257A/D257A^.

Lastly, Kaplan-Meier analysis of the survival data revealed that there was no statistical difference in survival between control diet and calorie-restricted D257A/D257A males or females (Fig 3). The survival data in Table 2 show no significant differences in the mean, median, 90%, or maximum survival between control diet and calorie-restricted D257A/D257A males or females, indicating that CR does not extend the lifespan of mitochondrial mutator mice.

**Table 2:** Survival parameters of PolgD257A/D257A mice on control and calorie-restricted diets.

	Females		Males	
	Control	CR	Control	CR
Number of subjects^a^	28 (7)	23 (7)	24 (5)	29 (9)
Mean (days)	470	443	442	463
Median (days)	487	460	473	482
90^th^ Percentile (days)	547	506	545	544
Maximum (days)	564	515	563	556

^a^Number of censored subjects is given in parentheses.

Mean survival is estimated from the area under the survival curves.

Median survival and 90th percentile values are derived from the Kaplan-Meier analysis and are the earliest timepoints at which the survival fraction is ≤0.5 or ≤0.1, respectively.

## Discussion

Point mutations and deletions in mtDNA accumulate with age in a variety of tissues [21], and a lifelong accumulation of such mutations in postmitotic tissues that demand high energy is thought to result in mitochondrial energy depletion, causing tissue dysfunction and eventually age-related pathologies. In agreement with this hypothesis, there are over 80 pathogenic mutations in the human *POLG* gene, and some of these are associated with Alper’s syndrome, PEO (progressive external ophthalmoplegia), or ataxia, and cause a variety of symptoms, including ophthalmoplegia, cataracts, hearing loss, progressive muscle weakness, parkinsonism, and cardiac dysfunction [22–23]. Mitochondrial mutator mice display a 10-90-fold increase in mtDNA deletions [13] and a >100-fold increase in point mutations in mtDNA in the heart and brain [14]. These mice display mitochondrial diseases and aging symptoms such as motor function decline, cardiac dysfunction and muscle mass loss, and hearing loss [20, 21, 23]. We have previously proposed that phenotypes of D257A/D257A mice are due to loss of somatic stem cells, since tissues displaying early onset phenotypes have high cellular turnover [11]. Recent studies have confirmed that D257A/D257A mice have a profound somatic stem cell defect [24–25]. Thus, CR failed to extend the healthspan or lifespan of D257A/D257A mice likely because the dietary intervention failed to rescue somatic stem cell loss or dysfunction in the context of damaged mitochondria in D257A/D257A mice.

It is thought that a central mechanism of aging retardation by CR is prevention of mitochondrial oxidative stress. In agreement with this hypothesis, CR decreases mtDNA damage and deletions in multiple tissues of aged rats [8–10], although no such effect was observed in primates [26]. CR also reduces age-related oxidative damage in the cochlea of mice by inducing SIRT3, a mitochondrial deacetylase [4]. Repression of the GH/IGF-1 axis has also been linked to extended lifespan in multiple species, and mouse models with reduced GH and/or IGF-1 display CR-like anti-aging effects, including reduced levels of mtDNA oxidative damage in the brain, reduced body size, and increased median lifespan [6]. In these long-lived dwarf mice, a defect in the GH/IGF-1 axis results in the activation of the transcription factor Foxo3a, which in turn binds to the promoter of the mitochondrial antioxidant enzyme *Sod2* (superoxide dismutase 2) gene that decomposes ROS in the mitochondria [27]. These observations are consistent with the idea that protection of mtDNA through the reduction of mitochondrial ROS may be a central mechanism by which CR reduces risks for age-related pathologies in mammals. During normal aging, mtDNA mutations or deletions in post-mitotic tissues are thought to be induced as a result of oxidative stress, since the level of such mutations in the heart and skeletal muscle of mice can be reduced by a mitochondrially targeted catalase transgene [20, 28]. In contrast, most mtDNA mutations that accumulate in D257A/D257A mice are thought to be due to mtDNA replication errors. Thus, a reduction of mitochondrial oxidative stress may do little to reduce the overall level of mtDNA mutations in D257A/D257A mice, explaining the lack of effect of CR on age-related parameters in this model. We note that although significant changes in a variety of oxidative stress markers have not been consistently detected in mitochondrial mutator mice [11, 29], D257A/D257A mice carrying a mCAT transgene did display prevention of age-related cardiac dysfunction [20], and a more recent analysis of mtDNA in POLG mice has revealed increased oxidative damage in muscle [30]. These observations are consistent with the proposed role of POLG in mitochondrial base excision repair, which is involved in the repair of oxidative damage to mtDNA [31]. Possibly, there may be a tissue-specific contribution of oxidative stress to this accelerated aging model, explaining the apparent lack of oxidative damage to mtDNA in some studies.

The current study suggests that the multiple health benefits of CR reported in normal animals may not be applicable to animals carrying gene mutations that lead to progeroid syndromes, such as mutations in genes involved in DNA repair or DNA metabolism [32]. This could be explained by the fact that there are profoundly different underlying aging mechanisms in progeroid mice as compared to normal animals. Alternatively, progeroid mice may have metabolic or mitochondrial alterations that preclude the establishment of the CR adaptations that lead to increased healthspan and lifespan observed in normal animals. For example, a central metabolic adaptation to CR is a decrease in insulin levels and increased insulin sensitivity. This CR effect is observed both in wild-type mice and in GH receptor knockout mice (GHRKO), which are long-lived and display further life extension under CR [33]. Alterations in key metabolic pathways including Akt/Foxo1, Sirt1 and Sirt3 may mediate these metabolic effects [4, 34]. Thus, future studies should try to characterize the metabolic response to CR in POLG mice. Interestingly, aerobic exercise is able to extend lifespan in POLG mice and to reduce aging phenotypes [35] and these effects are p53-mediated and may involve induction of repair mechanisms in mtDNA [36].
